# Treatment of Spreading or Diffuse Peritonitis1Read before the Medical Society of the County of Cattaraugus, December, 1909.

**Published:** 1910-06

**Authors:** Francis W. McGuire

**Affiliations:** Buffalo, N. Y.; Attending Surgeon Emergency Hospital


### Treatment of Spreading or Diffuse Peritonitis.[1]

By FRANCIS W. McGUIRE, M.D., Buffalo, N. Y.
Attending Surgeon Emergency Hospital.

SEPTIC peritonitis, one of the most important diseases that the surgeon is called upon to treat, is caused from infection of the peritoneum by any one or more of the various pathogenic microörganisms. A peritonitis may be local, spreading or diffuse, or general. Some writers have carelessly described the general for the spreading, as cases of genuine general peritonitis are rare, and I believe that the term spreading is more comprehensive and should be used instead, as it is impossible to tell how much of the peritoneum is involved. In order that we may be quite clear as to what is meant by spreading peritonitis, I had better state that spreading peritonitis is one in which more or less of the peritoneum is involved, in which there are no lymph barriers or adhesions walling off the infection. It is this type, I believe that interests us more, as the treatment of localised peritonitis has been definitely decided on general surgical principles.

The immediate dangers arising from peritonitis are sepsis, shock, pain, intestinal paresis, any one of which may be sufficient to cause death. Generally speaking, the danger is dependent on the virulence, dose of infection, resistance of the patient, area and, to some extent, the region involved. The treatment of this condition is based on our knowledge of its septic origin and the recognition of the fact that the chief danger consists of the absorption of toxic products, and means to provide for the elimination of these products from the most rational therapeutic indication.

It would seem that its treatment is essentially surgical, but that it is not necessarily so is manifested by the temporary and permanent recoveries that occasionally occur under medical treatment or under no treatment at all; but if we consider the morbidity of this condition, there will seem little question that modern surgery properly applied is a conservative form of treatment.

---

1. Read before the Medical Society of the County of Cattaraugus, December, 1909.

As to the locality infected, other things being equal, the higher the infection in the peritoneal cavity the greater the mortality, because of the more rapid and voluminous absorption through the large lymph spaces, and the great amount of peritoneal surface involved, while infections lower down are less so until we reach the pelvis, where the mortality is comparatively small. Our reason for this is found in the anatomy of the lymphatics and the return circulation of the abdominal cavity. According to Byron Robinson, the peritoneum is a large lymph sac and an infection of this membrane is a lymphangitis. In the diaphragmatic area the lymph channels are large and numerous. In the intestinal region the lymph channels are neither so large or numerous, while in the pelvis they are absent. This region is however rich in capillary lymphatics, hence infection of the pelvic peritoneum quickly occludes the small capillary channels and absorption is prevented, hence the septic process therefore, remains localised. On the other hand, the peritoneum in the intestinal region with its larger and less easily occluded lymph channels, when the infection furnishes septic products, allows of a greater absorption and therefore a greater toxicity. And like results follow infection of the diaphragmatic region, only with more rapid absorption. Authors differ as to whether the infection passes by way of the so-called stomata, or between the endothelial cells. But, be that as it may, experience has taught us that the absorption of septic products, whether by osmosis or cellular action, or both, are markedly active in the upper abdomen. This condition is of the utmost importance to the surgeon in the treatment of this disease, as it is his ally and a safeguard to the patient.

While considering the lymphatics, we might turn for a moment to the return circulation of the abdomen. We find the greater part of this circulation is emptied into the portal vein, there to be acted upon by the liver. From our knowledge of the physiology of the liver, we know that the greater part of the purification of the blood is accomplished in this organ, and it is an adjunct to the lymphatic glands in the destruction of microörganisms. But there is a portion of the return circulation of the stomach, also the return circulation of the diaphragm, which is not sent back through the liver. This condition would appear to have a bearing on the remarkable toxicity of infection in this region. It would seem that one of the essentials in the treatment of this condition would be the elevation of the diaphragmatic region, so to allow septic products to drain into the pelvis, for there by means of the omentum and pelvic colon sepsis may be controlled by absorption through the lymphatics and radicles of the portal vein.

The degree of elevation has a bearing on this condition, and has been worked out by Coffey. He took plaster-of-paris casts of the three basins of the abdomen, (right and left flanks and pelvis). He found that either flank would hold more fluid than the pelvis and is an inch deeper. The bottom of the right flank is four inches lower than the top of the divide made by the psoas muscle, on which the appendix is located. He found that in order to drain the fluid successfully by Fowler's position, that the body must be elevated to an angle of sixty or seventy degrees.

Before passing to the various treatments we might consider the question of how long the peritoneal cavity can be drained. Dr. J. L. Yates has confirmed the findings of other investigators, and his experiments seem to be more definite. He used dogs and was able to give quite definite times when drainage ceased. He inserted carmine solution at the ensiform cartilage, after placing different kind of drains in both flanks. He found that there was absolutely no drainage after six hours. It is quite evident, though apparently believed by but few, that the peritoneal cavity can not be drained for any great length of time, and if this be remembered by the surgeon, he will place his drainage with the idea of removing septic products in the shortest time, and will remove drainage materials much earlier than has hitherto been the custom.

Regarding the operative treatment of this condition, it may be divided into two classes:

1. Patients seen during the first twelve to thirty-six hours.

2. Patients seen after thirty-six hours.

1. Regarding those seen during the first thirty six hours (usually appendices), surgeons are unanimous that the time to operate is at once. The origin of the infection should be removed, an appendix should be nucleated, perforation should be closed, an abscess should be drained. These procedures should be done gently and with the least possible disturbance, as the inflamed peritoneum is easily injured and very susceptible to infection. Moist drains, if any at all, should be used. Irrigations, I believe, should not be used. The peritoneum will take care of a small amount of sepsis if the original focus is eradicated, and the wound may generally be closed with capillary drains to the parieties.

2. Those seen after thirty-six hours. There are three forms of treatment practised by American surgeons,—namely:

1. The "starvation treatment," also known as Ochsner's treatment.

2. The treatment outlined and followed by Blake and his adherents.

3. The method advanced and followed by Murphy of Chicago.

1. In the starvation treatment it is hoped for spontaneous subsidence, attenuation of the toxins, and localisation of the infection. The reason for using this method is founded upon the theory that peristalsis spreads infection. All food given by the mouth should be stopped, laxatives avoided, stomach should be washed thoroughly until it is free from the back flow from the upper intestine, nourishment is kept up by rectal nutrient enemata of about four ounces every four hours. This treatment is adapted for the most part, to inflamed gall-bladders and appendices. The use of this method in perforating ulcers, or strangulation, would seem to me to be very poor judgment. Ochsner maintains if this treatment is instituted early in the most violent and dangerous forms of acute gangrenous, or perforated appendicitis, that they will become mild and harmless. The application of this treatment, however, requires good surgical judgment, and should be in the hands of surgeons of experience, or be used possibly by practitioners who are not within easy reach of surgical help.

2. The method of Blake consists of removing the focus of infection, as closing of a perforating ulcer, removal of an appendix, and the like. The abdominal cavity should be thoroughly irrigated until the return flow remains clear. Closure of the peritoneum with parietal drainage. When the stomach is irrigated an ounce or two of Epsom salts is left in. Salines are given per rectum and the patient is placed in Fowler's position. In cases where infective necrotic foci cannot be removed, the peritoneum is drained.

3. Dr. Murphy, in the method of treatment advanced by him, seems to have eliminated harmful and unnecessary measures, and to have retained everything apparently necessary and good. He attributes his failures in the past to delay in operating, and to the fact that surgical intervention was either excessive or deficient. Excessive manipulations and washing of the intestine's leads to fresh and overpowering absorption of septic products. When the source of septic supply is not removed and drainage adopted, death frequently occurs from the continuance of the process.

He attributes his success to the following:

1. Elimination of the cause of the peritonitis.

2. Providing continual drainage—namely, through suprapubic opening and free drainage through the operative incision.

3. Avoiding manipulation, as sponging and washing the peritoneum, separating adhesions, and the like.

4. Rapid operation with a minimum of anesthesia.

5. Aiding the elimination of the poisons in the blood by introducing large quantities of saline solution in the rectum.

6. Elevation of the patient causing flow of septic material to the least absorptive zone of the perioneal cavity—namely, the pelvis.

7. Irrigation of the stomach when necessary.

In addition to the methods above oulined, there are numerous modifications of one kind or another. In cases where there is excessive gaseous extension of the gut, this viscus may be opened in one or more places and emptied of its contents.

In considering the different methods it would seem that the Ochsner treatment is almost impossible in children, on account of the difficulty of washing the stomach. That it has no place in the treatment of mild cases, I believe there is no question. That it has a place in the treatment of desperate cases I would not care to advocate. Personally, in those desperate cases where the patient cannot stand a general anesthetic, I would use local anesthesia and merely open and drain the peritoneal cavity, using the starvation treatment afterward.

Of the other two treatments (Blake and Murphy), it is surprising to note that the only difference is irrigation and closure of the abdomen in the one, and non-irrigation and drainage in the other. It seems that the most important procedures they both follow—namely, closure of the hole, rapid operation, Fowler's position, and salt solution per rectum. I believe that the methods followed by Murphy are the better, but the statistics of Blake would teach us that the abdomen can be closed oftener than has hitherto been the custom.

I believe that in women the culdesac should be drained instead of making a suprapubic stab wound. It has the advantage of placing the opening at the lowest portion of the peritoneal cavity, thereby facilitating drainage. It will frequently permit the anterior abdominal wound to close by primary union, besides avoiding the extraäbdominal incision.

It would seem that if the Fowler position is useful after operation, it might also be used to advantage before operation, and especially in cases of ruptured appendices, ruptured gall-bladders, and the like. If this position could be maintained previous to operation, it would have the advantage of allowing the infective materials to flow to the bottom of the pelvis, thereby lessening the general toxicity of the patient.

REFERENCES

Gray's Anatomy, p. 1264.
Mayo, Am. Jour. of Med. Scien., Jan., '07.
Coffey, Jour. Am. Med. Asso.
Yates, Jour. of Surg. Gyn. Obst., Dec.,'05.
A. N. M. Robinson, Lancet, Dec. 20, '06.
Munro, Keen's Surgery, Vol. III, p. 745,
Blake, N. Y. Med. Jour., '04.
LeConte, An. of Surg., '06.
Gibbon, N. Y., Med. Jour., '06.
Ochsner, Trans. Am. Surg. Asso., '02.
Murphy, Am. Jour. Obst.. '03.

470 FRANKLIN STREET.

